# Household-Level Vector-Borne Disease Prevention Practices and Healthcare Access Barriers in Rural Alta Verapaz, Guatemala: A Cross-Sectional Study

**DOI:** 10.7759/cureus.104307

**Published:** 2026-02-26

**Authors:** Mitchell M Michalak

**Affiliations:** 1 Department of Health Professions, Rollins College, Orlando, USA

**Keywords:** access to healthcare, indigenous health, observational cross-sectional study, rural guatemala, vector-borne diseases

## Abstract

Introduction

Vector-borne diseases (VBDs) remain endemic in Guatemala, including in Indigenous communities where access to preventive and healthcare resources may be limited. This cross-sectional study aimed to estimate the prevalence of household-level VBD prevention practices, transmission knowledge, healthcare access barriers, and self-reported syndromic illness among Indigenous Maya households in Alta Verapaz, Guatemala.

Methods

A cross-sectional household survey was conducted over five days in July 2025 across four mobile outreach clinic sites in Alta Verapaz. One adult representative per household (N = 111) completed a structured questionnaire assessing VBD prevention behaviors, transmission knowledge, healthcare access barriers, housing characteristics, and recent household febrile illness. Descriptive analyses were performed using R statistical software (R Foundation for Statistical Computing, Vienna, Austria).

Results

Preventive practices varied across clinic sites. A total of 91 (82.0%) households reported covering water containers, and mosquito net use ranged from eight (22.2%) to 19 (54.3%) households, depending on the clinic site. Additionally, 64 (57.7%) households reported taking no preventive action. Knowledge related to VBD transmission was generally low and varied across clinic sites. The most frequently reported barriers to healthcare access were distance to care for 56 (50.5%), lack of money for 41 (36.9%), and transportation barriers for 20 (18.0%) households. Recent household febrile illness with rash and joint pain was reported by 43 (38.7%) households; however, these findings were not laboratory confirmed.

Conclusions

This descriptive assessment identified variation in prevention practices and healthcare access barriers among Indigenous Maya households in Alta Verapaz. Rapid household surveys integrated into mobile outreach clinics may provide locally relevant information to support context-specific prevention planning.

## Introduction

Vector-borne diseases (VBDs), such as malaria, arboviral infections, and leishmaniases, account for approximately 17% of the global burden of infectious diseases [1]. Countries situated at tropical and subtropical latitudes report the greatest number of infections due to hospitable vector conditions [2]. Populations living in low- and middle-income countries (LMICs) experience a disproportionate burden of VBDs compared to those in high-income countries [2]. In many LMIC settings, robust surveillance systems, accessible healthcare for high-risk populations, and sustained funding for preventive services are limited or fragmented [3]. Globalization, climatic changes, population migration, and deforestation contribute to increased VBD incidence [4]. As a result, VBDs continue to cause substantial morbidity in tropical and subtropical regions [5].

Guatemala is among the poorest countries in Central America and frequently experiences elevated rates of VBDs each year [6]. Recent epidemiologic trends further underscore ongoing transmission. According to an epidemiological report from the Pan American Health Organization [7], there was a five-fold increase in dengue fever cases in Guatemala from the spring and summer months of 2023 to 2024 during the same seasonal period. Similarly, from 2011 to 2019, Guatemala reported a 98% increase in cutaneous leishmaniasis cases [8]. These trends highlight ongoing VBD activity in Guatemala, including in rural regions [9].

Indigenous Maya populations comprise approximately 40% of the national census [10] and represent the most impoverished demographic group in Guatemala. Indigenous Maya communities, especially those living in the rural highlands of Alta Verapaz, experience health disparities driven by poverty [11], geographic isolation, linguistic barriers, and limited access to healthcare services [9]. Structural factors such as inadequate housing and sanitation [5], combined with residential proximity to vector breeding sites [12], further increase transmission risk. Barriers to healthcare access may limit detection of VBDs in some Indigenous Maya communities [9].

Despite elevated risk, limited evidence exists describing household-level VBD prevention practices, perceptions, and feasibility [13] of preventive behaviors among rural Indigenous communities in Guatemala [14]. Characterization of local preventive practices and existing VBD knowledge may help inform future disease prevention campaigns and reduce ongoing transmission. This study aimed to describe household-level VBD prevention practices, knowledge, and healthcare access barriers among rural households in Alta Verapaz, Guatemala.

## Materials and methods

Study design

This study utilized a cross-sectional household survey conducted over five days in late July of 2025. Questionnaires were administered to one adult representative per household, primarily mothers or fathers. This study spanned four different clinic sites and was conducted in collaboration with two community-based medical service organizations (CBOs) providing field clinical services at common community locations. All field clinic sites and study locations were within the central and northern regions of Alta Verapaz. The study population consisted of Maya communities that primarily spoke either Q’eqchi’, K'iche', or Poqomchi’. Surveys were administered in Spanish or local Mayan languages with the assistance of multilingual field staff. The questionnaire was pilot tested within a similar Indigenous population approximately three months prior to data collection. Feedback from the pilot testing informed minor revisions to question framing and wording to improve clarity before field implementation.

Geographic and cultural setting

Alta Verapaz is one of the most populous departments in Guatemala and contains a high proportion of Indigenous Maya residents [15]. The department is located in the north-central region of the country and is largely comprised of tropical rainforest and the Sierra de Las Minas Mountain range. The mountainous terrain and dispersed settlement patterns strongly influence access to healthcare services for many communities [16].

Many households rely on subsistence farming or migrant labor for financial support [17], a pattern common among rural and Indigenous Guatemalan populations. Limited Spanish proficiency has been widely documented among Poqomchi’ and other Mayan linguistic groups and may influence health-seeking behavior and healthcare access [16]. In addition to communication challenges, geographic isolation and reliance on traditional health practices may further influence healthcare utilization and illness reporting [18]. These contextual characteristics informed survey development, administration, and interpretation.

Sampling

This study employed convenience sampling and prioritized adult household representatives present at the CBO field clinics. Clinic providers informed all households seeking care about the study and the option to participate. Participation was voluntary and did not affect access to clinical services. Inclusion criteria were adults aged 18 years or older who resided in the household, self-identified as Indigenous, and consented to participate. The only exclusion criteria were individuals under 18 years of age, those who did not self-identify as Indigenous persons, and those who declined to participate in the study.

Data collection

Data were collected using a structured household survey. Survey questions were developed for this study, culturally tailored to the local context, and were not directly adapted from previously published instruments. The questionnaire was designed to prioritize brevity and feasibility within a mobile outreach clinic setting. Formal psychometric validation was not performed; however, pilot testing assessed clarity, cultural relevance, and comprehension. The survey assessed responses across six domains: prevention practices, household demographics, VBD transmission knowledge, housing characteristics, barriers to healthcare, and self-reported febrile illness among any household member. The questionnaire consisted of nine questions with multiple-choice selections and two free-response questions regarding household size and the number of household members under the age of five. Surveys were administered in person by trained field staff using standardized data collection forms; live verbal interpretation between Spanish and the local Mayan languages was facilitated as necessary. Returned surveys were reviewed for completeness prior to data entry. The full survey questionnaire used for data collection is included in the Appendices.

Data analysis

Responses were recorded on paper forms and entered into a Microsoft Excel spreadsheet (Microsoft Corporation, Redmond, WA) for data storage and cleaning before importing into R statistical software version 4.4.2 (R Foundation for Statistical Computing, Vienna, Austria). Responses of “sometimes” and “yes” were classified as affirmative responses, while “no” and “I don’t know” were classified as negative responses to facilitate descriptive comparison. Descriptive statistics, cross-tabulated summaries, and data visualizations were generated using R with standard statistical and visualization packages. Because this study was designed to provide descriptive prevalence estimates rather than identify independent risk factors, no hypothesis testing or inferential statistics were performed.

Ethics statement

This study was reviewed and approved by the Institutional Review Board of Rollins College (Approval No. 20250507MM). Verbal informed consent was obtained from all participants prior to survey administration. Participation was voluntary and did not affect access to clinical services. No personal identifiers were collected. This study was conducted in collaboration with local CBOs facilitating clinic operations.

## Results

Descriptive characteristics and survey findings

A total of 111 respondents were surveyed across four clinic sites, with each respondent representing one household. The mean household size was 6.0 (SD = 2.7), and 77 (69.4%) households reported having at least one child under five years of age (Table 1).

**Table 1:** Demographics and household characteristics by clinic site. Percentages reflect recoded responses in which “Yes” and “Sometimes” were classified as affirmative.

		Demographics			Housing characteristics			
Clinic	N	Household members, Mean (SD)	Children <5, Mean (SD)	Households with children <5, n (%)	Dirt floor, n (%)	Cement floor, n (%)	Metal roof, n (%)	Thatched roof, n (%)
Clinic 1	35	5.0 (1.7)	1.1 (0.9)	24 (68.6%)	24 (68.6%)	9 (25.7%)	26 (74.3%)	1 (2.9%)
Clinic 2	36	6.7 (3.3)	1.4 (1.2)	28 (77.8%)	29 (80.6%)	2 (5.6%)	26 (72.2%)	2 (5.6%)
Clinic 3	20	7.0 (3.2)	1.0 (0.8)	15 (75.0%)	19 (95.0%)	0 (0.0%)	18 (90.0%)	3 (15.0%)
Clinic 4	20	5.5 (2.0)	1.0 (1.7)	10 (50.0%)	16 (80.0%)	1 (5.0%)	17 (85.0%)	1 (5.0%)
Total	111	6.0 (2.7)	1.2 (1.2)	77 (69.4%)	88 (79.3%)	12 (10.8%)	87 (78.4%)	7 (6.3%)

**Figure 1:**
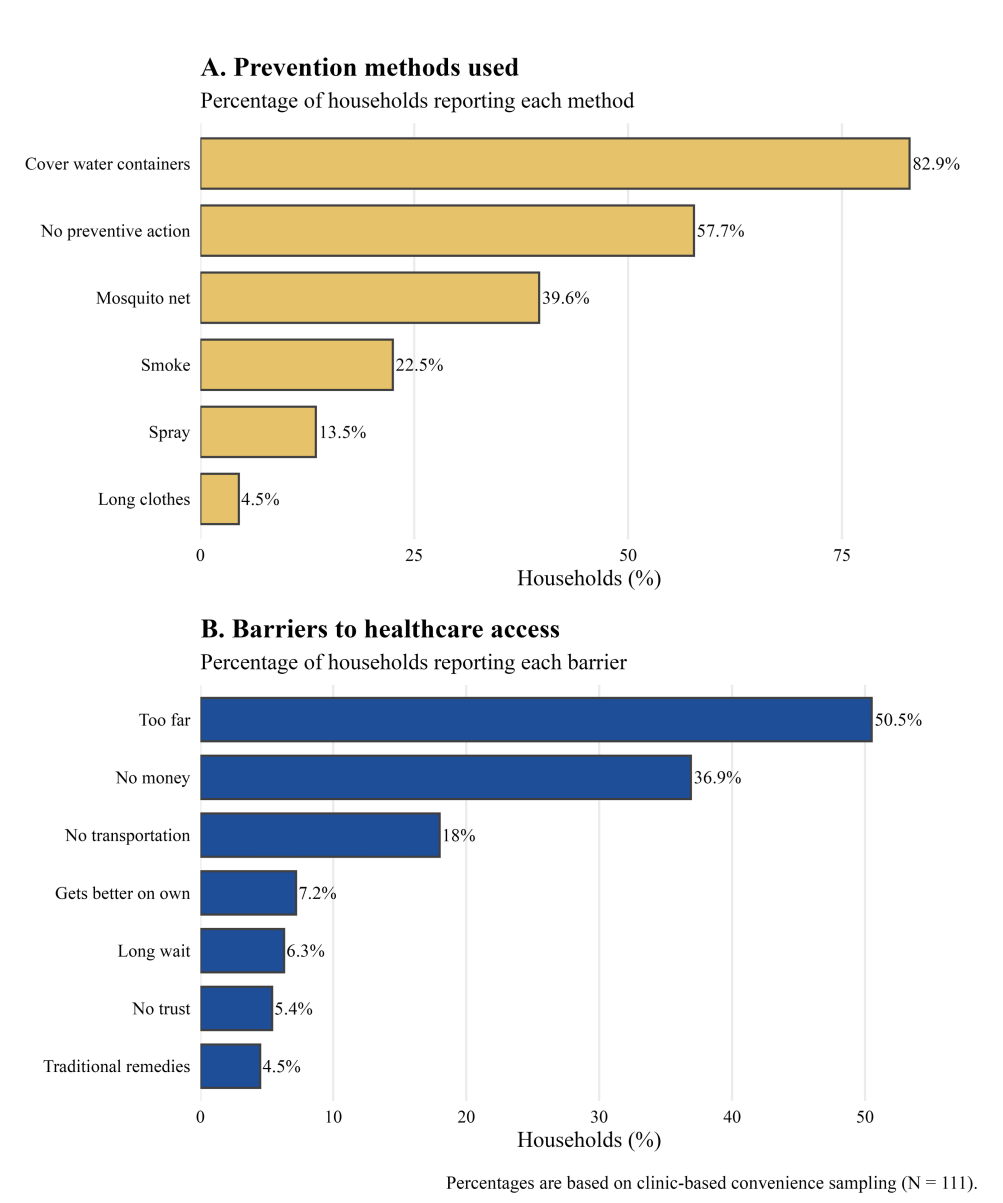
Prevention practices and barriers to healthcare access among households.

Reports of VBD preventive behaviors were mixed (Figure 1). Overall, mosquito net use was reported by 44 (39.6%) households, while 64 (57.7%) reported no preventive action. In contrast, 91 (81.9%) households reported covering water containers. Other prevention behaviors were less common, including insecticide spraying by 15 (13.5%), use of smoke by 25 (22.5%), and wearing long protective clothing by five (4.5%) (Table 2). Preventive behaviors such as mosquito net use varied by clinic site, ranging from eight (22.5%) to 19 (54.3%).

**Table 2:** Survey findings by clinic site. Each clinic day represents a distinct clinic location. Percentages reflect recoded responses in which “Yes” and “Sometimes” were classified as affirmative. Respondents had the option to select all responses that apply.

	Overall, n (%)	Clinic 1, n (%)	Clinic 2, n (%)	Clinic 3, n (%)	Clinic 4, n (%)
Preventive behaviors					
Covering water containers	91 (82.0%)	27 (77.1%)	32 (88.9%)	18 (90.0%)	14 (70.0%)
No preventive action	64 (57.7%)	16 (45.7%)	25 (69.4%)	12 (60.0%)	11 (55.0%)
Mosquito net use	44 (39.6%)	19 (54.3%)	8 (22.2%)	7 (35.0%)	10 (50.0%)
Use of smoke	25 (22.5%)	8 (22.9%)	9 (25.0%)	3 (15.0%)	5 (25.0%)
Insecticide spraying	15 (13.5%)	6 (17.1%)	1 (2.8%)	5 (25.0%)	3 (15.0%)
Wearing long clothing	5 (4.5%)	3 (8.6%)	1 (2.8%)	0 (0.0%)	1 (5.0%)
Barriers to healthcare access					
Distance to care (too far)	56 (50.5%)	12 (34.3%)	19 (52.8%)	10 (50.0%)	15 (75.0%)
Lack of money	41 (36.9%)	12 (34.3%)	19 (52.8%)	4 (20.0%)	6 (30.0%)
No transportation	20 (18.0%)	3 (8.6%)	8 (22.2%)	5 (25.0%)	4 (20.0%)
Long wait times	7 (6.3%)	5 (14.3%)	0 (0.0%)	1 (5.0%)	1 (5.0%)
Lack of trust in healthcare	6 (5.4%)	5 (14.3%)	0 (0.0%)	0 (0.0%)	1 (5.0%)
Health-seeking beliefs					
Gets better on its own	8 (7.2%)	3 (8.6%)	1 (2.8%)	3 (15.0%)	1 (5.0%)
Traditional remedies	5 (4.5%)	4 (11.4%)	0 (0.0%)	1 (5.0%)	0 (0.0%)
Knowledge					
Vector-borne disease knowledge	34 (30.6%)	12 (34.3%)	4 (11.1%)	8 (40.0%)	10 (50.0%)
Recent illness					
Recent fever with rash and joint pain	43 (38.7%)	10 (28.6%)	18 (50.0%)	7 (35.0%)	8 (40.0%)

**Figure 2:**
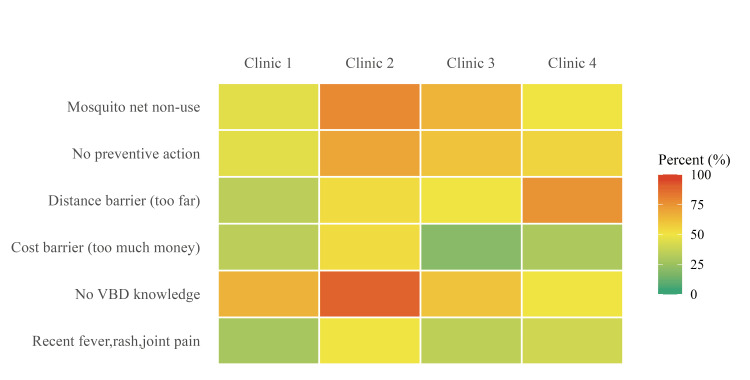
Vector-borne disease prevention practices and healthcare barriers by clinic site. Respondents had the option to select all responses that apply. VBD: Vector-borne disease.

Healthcare access barriers were frequently reported. Distance to care ranged from 12 (34.3%) to 15 (75.0%), and lack of money ranged from four (20.0%) to 19 (52.8%). Long wait times ranged from 0 (0.0%) to five (14.3%), and lack of trust in healthcare services ranged from 0 (0%) to 16 (14.3%). Knowledge related to VBDs differed across clinic sites but was generally low, ranging from four (11.1%) to 10 (50.0%). Recent household febrile illness with rash and joint pain was reported by 10 (28.6%) to 18 (50.0%) households. Variation across clinic sites was observed in preventive practices, healthcare barriers, and self-reported febrile illness (Figure 2).

## Discussion

This cross-sectional assessment identified variation in household-level VBD prevention practices and common healthcare access barriers among Indigenous Maya households served by mobile outreach clinics in Alta Verapaz [5,9]. While low-cost behaviors such as covering water containers were commonly reported, most households reported no preventive actions. Knowledge related to VBD transmission remained low across clinic sites, a pattern similarly observed in other community-based studies conducted in Guatemala [13]. Given the frequency of reported febrile illness with rash and joint pain, further follow-up investigation may be warranted in settings with limited diagnostic access [3]. The observed pattern of preventive behaviors may reflect differences in resource availability; however, causal interpretation cannot be determined within this cross-sectional design. While water container coverage represents a low-resource behavior that can be adopted without external support, mosquito nets, insecticide spraying, and protective clothing require financial investment or sustained availability [5]. The limited uptake of these measures suggests that resource-intensive prevention strategies may be less feasible without programmatic support and resource alignment.

The most frequently reported barrier to healthcare access was distance to health facilities, followed by financial and transportation constraints. These barriers reflect structural determinants of health rather than individual reluctance to seek care, a pattern widely documented among Indigenous Maya populations in Guatemala [18]. The relatively low reporting of distrust in healthcare services or long wait times suggests that limited utilization is driven primarily by geographic and economic inaccessibility rather than behavioral factors.

In geographically isolated settings with limited fixed healthcare infrastructure, mobile outreach clinics can support service delivery and prevention messaging. These clinics may also provide situational awareness and early identification of syndromic signals [3]. Embedding rapid household-level assessments within routine outreach activities may enable real-time adaptation of prevention strategies and improved targeting of underserved areas.

Limited published data exist that describe household-level VBD prevention practices in these specific communities. Because Indigenous Maya communities may experience barriers to healthcare access outside of mobile outreach clinics, they are unlikely to be captured in routine surveillance data, complicating accurate burden estimation [3]. Early symptom recognition and prompt diagnostic testing remain limited in some of these settings [9].

Rapid household-level assessments offer a practical approach for generating timely, program-relevant data in surveillance-limited settings [3]. In areas with sparse routine data, such assessments can provide baseline insights into community knowledge and preventive behaviors, allowing programs to more efficiently identify gaps and tailor interventions.

Part of this assessment included a proxy measure of recent household illness characterized by fever, rash, and joint pain. Although self-reported, nonspecific, and not laboratory confirmed, syndromic reports may be sensitive but are not specific for arboviral disease based on Pan American Health Organization clinical definitions [7]. When interpreted alongside epidemiological risk factors, such signals may serve as indicators of recent transmission in settings where diagnostic access is limited [3].

This assessment was designed as a pragmatic field-based evaluation embedded within mobile clinic operations. In surveillance-limited environments, rapid descriptive data collection may serve as a preliminary step prior to more rigorous epidemiologic investigations incorporating probability sampling or laboratory confirmation.

Limitations

This study had several limitations. Convenience sampling limited generalizability beyond participating households. No formal sample size calculation was performed, as the study was designed as a rapid, clinic-embedded assessment conducted over a defined outreach period. The sample size, therefore, reflects operational feasibility rather than statistical power considerations. Additionally, the study was not designed to identify independent risk factors or test hypotheses, limiting inferential interpretation. Responses were self-reported and subject to recall and social desirability bias. Syndromic illness was not laboratory confirmed. The cross-sectional design precluded causal inference. Findings should therefore be interpreted as descriptive and exploratory.

Despite these limitations, the findings provide descriptive insight for local decision-making and intervention planning. In settings where Indigenous communities are underrepresented in routine health data systems [18], rapid household assessments can inform prioritization of prevention strategies and guide development of more targeted future studies [9]. Future research should incorporate longitudinal designs, entomological or laboratory data, and culturally tailored interventions addressing structural determinants of VBD risk [5].

## Conclusions

This descriptive cross-sectional assessment identified variation in household-level VBD prevention practices and healthcare access barriers among Indigenous Maya households in rural Alta Verapaz, Guatemala. While low-cost preventive behaviors, such as covering water containers, were commonly reported, additional preventive measures were less frequent, and geographic and financial barriers to care were frequently described. Reports of recent febrile illness with rash and joint pain were observed, but were not laboratory confirmed.

Integrating brief household surveys into mobile outreach services may provide locally relevant information to support targeted prevention planning in resource-limited settings. These findings may serve as a baseline reference for future community-based assessments and more rigorous studies incorporating longitudinal follow-up or laboratory confirmation.

## Figures and Tables

**Figure 3:**
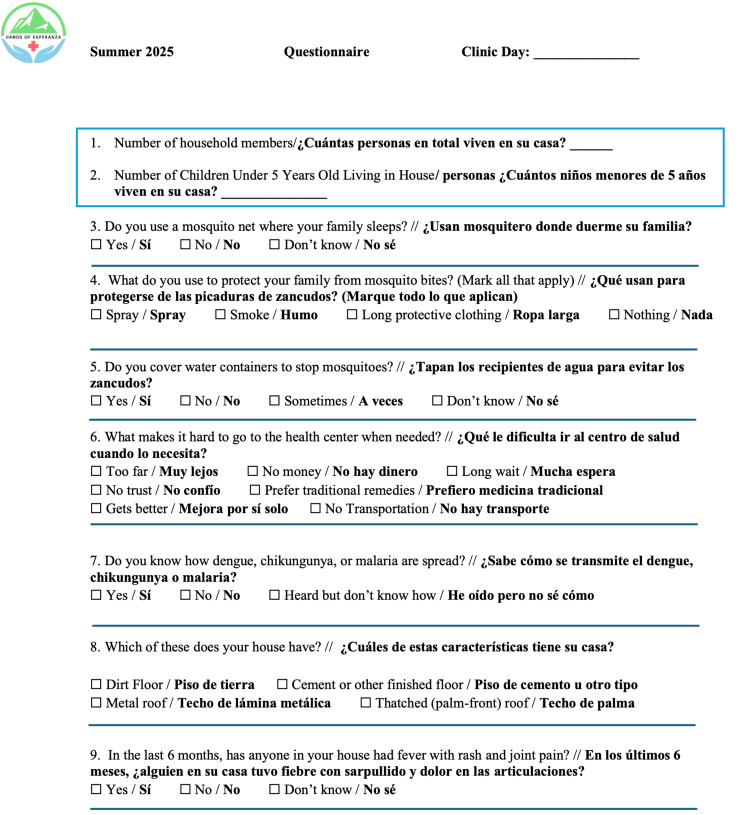
Vector-borne disease prevention and healthcare access questionnaire 2025.
